# Uteroplacental insufficiency leads to hypertension, but not glucose intolerance or impaired skeletal muscle mitochondrial biogenesis, in 12-month-old rats

**DOI:** 10.14814/phy2.12556

**Published:** 2015-09-28

**Authors:** Melanie Tran, Margaret E Young, Andrew J Jefferies, Deanne H Hryciw, Michelle M Ward, Erica L Fletcher, Mary E Wlodek, Glenn D Wadley

**Affiliations:** 1 Departments of Physiology, The University of MelbourneParkville, Victoria, Australia; 2 Departments of Anatomy and Neuroscience, The University of MelbourneParkville, Victoria, Australia; 3 School of Exercise and Nutrition Sciences, Centre for Physical Activity and Nutrition Research, Deakin UniversityBurwood, Victoria, Australia

**Keywords:** Aging, development, fetal programming

## Abstract

Growth restriction impacts on offspring development and increases their risk of disease in adulthood which is exacerbated with “second hits.” The aim of this study was to investigate if blood pressure, glucose tolerance, and skeletal muscle mitochondrial biogenesis were altered in 12-month-old male and female offspring with prenatal or postnatal growth restriction. Bilateral uterine vessel ligation induced uteroplacental insufficiency and growth restriction in offspring (Restricted). A sham surgery was also performed during pregnancy (Control) and some litters from sham mothers had their litter size reduced (Reduced litter), which restricted postnatal growth. Growth-restricted females only developed hypertension at 12 months, which was not observed in males. In Restricted females only homeostasis model assessment for insulin resistance was decreased, indicating enhanced hepatic insulin sensitivity, which was not observed in males. Plasma leptin was increased only in the Reduced males at 12 months compared to Control and Restricted males, which was not observed in females. Compared to Controls, leptin, ghrelin, and adiponectin were unaltered in the Restricted males and females, suggesting that at 12 months of age the reduction in body weight in the Restricted offspring is not a consequence of circulating adipokines. Skeletal muscle PGC-1*α* levels were unaltered in 12-month-old male and female rats, which indicate improvements in lean muscle mass by 12 months of age. In summary, sex strongly impacts the cardiometabolic effects of growth restriction in 12-month-old rats and it is females who are at particular risk of developing long-term hypertension following growth restriction.

## Introduction

In Western societies, uteroplacental insufficiency is the major cause of intrauterine growth restriction and is characterized by reduced uteroplacental perfusion of nutrients and oxygen delivery to the developing fetus (Bernstein et al. 2000; Haggarty et al. 2002). While the majority of epidemiological evidence supports the hypothesis that individuals born of low birth weight have increased susceptibility to developing cardiovascular and metabolic diseases (Barker et al. 1989, 1993; Hales et al. 1991; Barker 2006), the underlying mechanisms are poorly understood. Suboptimal conditions in utero alter the development of fetal organ systems which results in permanent changes in tissue structure, gene expression patterns, and physiological function that may be contributing to the common adult phenotypes described (Hoy et al. 1999; Simmons et al. 2001; Wlodek et al. 2007). Impairments in renal function and skeletal muscle mitochondrial function have previously been implicated in various disease states (Kelley et al. 2002; Morino et al. 2005; Schreuder et al. 2007), providing an important mechanistic link to cardiometabolic outcomes associated with being born small. Furthermore, in these growth-restricted individuals, early postnatal growth independently predicts adult disease risk such that catch-up growth in early childhood often provides long-lasting benefits, in contrast to the detrimental effects of late accelerated growth (Eriksson et al. 2001). The mechanism for the rapid catch-up growth and the risk of developing obesity in adulthood is unclear. However, it may include aspects of enhanced orexigenic or reduced anorexic mechanisms modulated by the abnormal regulation of appetite-regulating hormones such as leptin, ghrelin, and adiponectin (Desai et al. 2005).

To allow for an appropriate control for growth-restricted animals several studies reduce control litter sizes after birth to avoid cofounders which may arise due to the different sized litters. However, we have previously reported that reducing litter size from sham-operated dams impairs maternal mammary morphology, lactation, and subsequent postnatal growth and health of the offspring (O’Dowd et al. 2008; Wadley et al. 2008; Wlodek et al. 2008) which may independently program later adverse outcomes. Therefore, Reduced litter offspring are not appropriate controls to the Restricted group, but rather an additional experimental group that allows for investigation on the impact of postnatal growth restriction on later health (Wadley et al. 2008; Wlodek et al. 2008). Increasing evidence has also suggested that the same prenatal insult can program sexually dimorphic differences with evidence supporting a greater phenotype in males compared with females, which may explain, in part, why females are often less studied (Simmons et al. 2001; Styrud et al. 2005; Denton and Baylis 2007; Grigore et al. 2008; Nusken et al. 2008; Mercuro et al. 2010).

Growth-restricted male rats, by uteroplacental insufficiency surgery, have low nephron endowment and become hypertensive by 6 months of age (Wlodek et al. 2008). Furthermore, these growth-restricted male rats have impaired metabolic control at 6 months (altered insulin sensitivity and glucose intolerance) which is associated with reduced pancreatic *β*-cell mass (Wlodek et al. 2007; Siebel et al. 2008, 2010; Laker et al. 2011) and skeletal muscle mitochondrial biogenesis markers (PGC-1*α*, COX IV, and Tfam) (Wadley et al. 2008). In contrast, growth-restricted females, by uteroplacental insufficiency surgery, have reduced nephron number and modest renal insufficiency at 6 months but do not develop hypertension (Moritz et al. 2009; Gallo et al. 2012b). Additionally, these growth-restricted females at 6 months have reduced basal hepatic insulin sensitivity and reduced *β*-cell mass, but demonstrate normal glucose control and unaltered markers of skeletal muscle biogenesis (Siebel et al. 2008; Wadley et al. 2008; Gallo et al. 2012b). Reducing litter size to five pups at birth also results in male hypertension at 9 and 22 weeks of age which is associated with fewer glomeruli at 6 months (Wlodek et al. 2008). Furthermore, offspring from Reduced litters have decreased skeletal muscle mitochondrial biogenesis and display mild insulin resistance at 6 weeks (Wadley et al. 2008; Laker et al. 2011) which may predispose them to future cardiovascular and diabetes risk.

Of interest, there is a clear relationship between altered glucose tolerance and retinal function, especially in disease states such as diabetes mellitus. The most common complication of diabetes is diabetic retinopathy which is the leading cause of preventable blindness, whose early clinical sign is retinal neurodegeneration (Bogdanov et al. 2014). In support of this link, streptozotocin-treated rats and diabetic db/db mice, whose plasma glucose concentrations are elevated, develop retinal neural dysfunction (Bearse et al. 2006; Phipps et al. 2007). Growth restriction impairs glucose tolerance and increases the risk of developing diabetes (Simmons et al. 2001; Styrud et al. 2005; Nusken et al. 2008). However, despite this, no studies at this time have characterized retinal function in offspring born small.

Although there is a clear interaction between prenatal exposure and postnatal environment, some developmental programming outcomes do not become apparent until well into the aging period or after being challenged with additional lifestyle insults in postnatal life. Low nephron endowment and *β*-cell deficits, for example, may not be sufficient to cause renal dysfunction, hypertension, or impaired glucose tolerance which is adequately compensated for at least until a postnatal stressor or “second hit” reveals a clinically relevant phenotype (Nenov et al. 2000; Moritz and Bertram 2006; Gallo et al. 2012b). Of particular interest to this study, aging has been associated with a decline in glucose tolerance, insulin secretion and sensitivity (DeFronzo 1981; Reaven et al. 1983; Chen et al. 1988), and renal function (Nishimura et al. 1988; Escriva et al. 1997; Baylis and Corman 1998), which may be exacerbated in susceptible offspring born small. In addition, as hypothalamic control of appetite is likely to be set during early development, alterations in nutrient availability may contribute to the development of adult obesity (Desai et al. 2005). Therefore, the aim of this study was to examine the effects of uteroplacental insufficiency and reducing litter size on cardiometabolic function in aging male and female offspring at 12 months. We hypothesized that aging to 12 months exacerbates the known adverse cardiometabolic function in growth-restricted male and female rats leading to hypertension and impaired glucose tolerance which is associated with a dysregulation of appetite-regulating hormones (leptin, ghrelin, and adiponectin), a decline in renal function and a reduction in skeletal muscle mitochondrial biogenesis. In view of the known affects that hyperglycemia has on retinal function, we also predicted that retinal function would be impaired (Fletcher et al. 2007; Phipps et al. 2007; Ly et al. 2011).

## Materials and Methods

### Animal procedures

All experiments were approved by the University of Melbourne Animal Ethics Committee prior to commencement and conducted in accordance with accepted standards of humane care. Wistar-Kyoto rats were housed in an environmentally controlled room (constant temperature 22°C) with a 12-h light–dark cycle and had access to food and tap water ad libitum. Female rats were mated at 18–24 weeks of age and surgery was performed on day 18 of pregnancy (Wlodek et al. 2005, 2007). Briefly, F0 pregnant rats were randomly allocated to a sham (offspring termed Control or Reduced) or uteroplacental insufficiency (offspring termed Restricted) group and were anesthetized with 4% isoflurane and 650 mL/min oxygen flow (reduced to 3.2% isoflurane and 250 mL/min oxygen flow when suturing). Uteroplacental insufficiency was induced by bilateral uterine vessel (artery and vein) ligation which restricts blood supply and nutrient delivery to the fetuses. The F0 female rats delivered naturally at term on day 22 of pregnancy and pups remained with their original mothers after birth and throughout lactation to eliminate the confounding factors associated with cross fostering. At birth, sham litters were allocated into either a control group with unaltered litter size (Control) or a reduced litter group (Reduced), where litter size was randomly reduced to five pups to match that of the Restricted litters (O’Dowd et al. 2008; Wadley et al. 2008; Wlodek et al. 2008). F1 offspring body weights were measured at postnatal days (PN) 1, 7, 14, and 35 and at 2, 3, 4, 6, 9, and 12 months (1 randomly allocated male and female from each litter; *n *=* *12–15 per group). Absolute and fractional growth rates were calculated in F1 offspring between PN14 and 2 month to assess growth rate during the peripubertal period (Tran et al. 2013).

### Blood pressure and 24-h renal function measurements

Systolic blood pressure was measured in the morning by tail cuff in animals that were acclimatized to the restraint procedure at 2, 4, 6, 9, and 12 months of age (Wlodek et al. 2008; Moritz et al. 2009; Gallo et al. 2012a,b). At 12 months of age, animals were weighed and placed individually in metabolic cages for 24-h measurements of food and water intake and urine production (Moritz et al. 2009; Gallo et al. 2012a, 2013). Urinary measurements of sodium, chloride, potassium (Rapidchem 744, Bayer Healthcare, CA), glucose, creatinine, albumin, and total protein (Cobas Integra 400; Roche Diagnostics, Burgess Hill, UK) were performed.

### Intraperitoneal glucose tolerance test and insulin challenge

At 11.5 months, an intraperitoneal glucose tolerance test was performed following an overnight fast. Tail vein blood samples (300 *μ*L) were taken prior to (–10 min and –5 min) and following an intraperitoneal bolus injection of 50% (wt/vol) glucose (1 g/kg body weight; Pharmalab, Lane Cove, NSW, Australia) at 5, 10, 20, 30, 45, 60, and 90 min (Laker et al. 2011; Tran et al. 2012). At 12 months of age, an insulin challenge was performed following an overnight fast to assess whole body insulin sensitivity. Tail vein blood samples were taken prior to and following a subcutaneous bolus injection of insulin (1 U/kg body weight; Actrapid, Novo Nordisk Pharmaceuticals, North Rocks, NSW, Australia) at 20, 40, and 60 min (Tran et al. 2012, 2013). At completion of the IPGTT and IC experiment, animals were allowed access to food and water ad libitum.

Plasma insulin concentrations were measured in duplicate using a commercially available rat insulin radioimmunoassay (RIA) kit as per manufacturer’s instructions (Millipore Corporation, Billerica, MA) (Tran et al. 2012, 2013). Plasma glucose concentrations were measured in duplicate using a scaled-down version of the enzymatic fluorimetric analysis (Laker et al. 2011; Tran et al. 2012, 2013). Plasma leptin (R&D Systems), ghrelin (Abnova), and adiponectin (R&D Systems) concentrations were measured using an enzyme-linked immunoassay assay (ELISA) as per the manufacturer’s instructions. Glucose and insulin area under curve (AUC) was calculated as the total area under curve from basal to 90 min for the IPGTT and from 0 to 60 min for the IC using the trapezoidal model (Martin et al. 2007). Homeostatic model assessment for insulin resistance (HOMA-IR) was calculated using the following formula: fasting plasma insulin (*μ*U/mL) × fasting plasma glucose (mmol/L)/22.5 (Matthews et al. 1985; Siebel et al. 2008; Laker et al. 2011; Tran et al. 2012, 2013).

### Retinal function in male offspring

Retinal function was assessed using the flash electroretinogram (ERG) (Phipps et al. 2007; Weymouth and Vingrys 2008; Ly et al. 2011). Briefly, following dark adaptation overnight rats (*n *=* *10 per group) were anesthetized with a mixture of ketamine and xylazine (60:5 mg/kg), corneas anesthetized with topical 0.5% proxymetacaine (Alcaine Allergan, Frenchs Forest, NSW, Australia), and pupils dilated with 0.5% tropicamide (Mydriacyl; Allergan, Frenchs Forest, NSW, Australia). Full-field flash ERGs were recorded with custom made AgCl recording electrodes placed on the central cornea, and references to a stainless steel ground (26 gauge needle) inserted in the tail. A commercial photographic flash unit (Mecablitz 60CT4) was used to generate a light stimulus that was delivered through a Ganzfeld sphere. The stimulus energy was attenuated by altering the flash aperture settings and implementing neutral density filters (1.5–2.1 log cd s/m^2^). A paired-flash protocol was utilized to isolate cone and rod contributions of the ERG waveform as described in Phipps et al. (2007). Two flashes were presented in succession with an interstimulus interval (ISI) of 0.8 sec, and the rod and cone responses isolated by digital subtraction.

The flash ERG generates a serial waveform consisting of an a-wave, b-wave, and oscillatory potentials reflecting the function of photoreceptors (a-wave) and inner retinal neurons (b-wave and OPs). In order to evaluate the effect of uteroplacental insufficiency on the function of these different classes of retinal neurons, the waveforms generated by the ERG was modeled as previously described (Phipps et al. 2007; Ly et al. 2011). The rod a-wave, which is referred to as the PIII when modeled, reflects photoreceptoral function and was measured using a modified computational description of the phototransduction cascade as described by the equation:

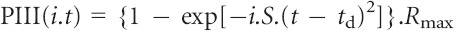

where PIII gives the summed photocurrent as a function of luminous exposure *i* (cd s/m^2^) and time, *t* (in seconds). *R*_max_ (microvolts) is the saturated amplitude of the PIII, whereas *S* (sensitivity) represents the gain of the phototransduction process (m^2^/cd/s^3^) and *t*_d_ (seconds) is a brief delay that accounts for biochemical and recording latencies following stimulation. The PIII model was fitted to an ensemble of a-waves (1.3–2.0 log cd s/m^2^) for each individual animal through the optimization of the *R*_max_, sensitivity, and *t*_d_ parameters. Optimization was accomplished through minimization of the sum of squares (SS) of error term using the solver function of Excel^–^.

The b-wave when modeled is called the PII and was derived by digitally subtracting the modeled photoreceptoral PIII from the raw waveform. The oscillatory potentials (OPs) were then filtered so that the PII could be described in terms of its amplitude and implicit time (time to peak in seconds). OPs were isolated by fitting an inverted gamma function to the rising slope of the PII and digitally subtracting this curve from the raw waveform. Three major OP peaks were isolated and described in terms of their amplitude and implicit time, and summed to gauge an overall effect on the OPs.

### Postmortem and tissue collection

Approximately 2 weeks after all experimental procedures, a postmortem were performed in male and female rats at 12 months of age. Nonfasted rats were anesthetized with an intraperitoneal injection of ketamine (100 mg/kg body weight; Parnell Laboratories, Alexandria, NSW, Australia) and Illium Xylazil-20 (30 mg/kg body weight; Troy Laboratories, Smithfield, NSW, Australia) and a cardiac puncture was performed. Heart, kidneys, gastrocnemius muscle, liver, pancreas, dorsal fat, and brain were excised, weighed, and then frozen in liquid nitrogen and stored at –80°C.

### Protein extraction and immunoblotting

Frozen gastrocnemius muscle was homogenized in freshly prepared ice-cold lysis buffer (10 *μ*L buffer/mg muscle; 50 mmol/L Tris buffer at pH 7.5 containing 1 mmol/L EDTA, 10% [vol/vol] glycerol, 1% [vol/vol] Triton X-100, 5 mmol/L sodium pyrophosphate [Na_4_P_2_O_7_], 50 mmol/L sodium fluoride, 1 mmol/L phenylmethylsulfonylfluoride, 1 mmol/L dithiothreitol, and 5 *μ*L/mL protease inhibitor cocktail; Sigma-Aldrich, Castle Hill, NSW, Australia) (Wadley and McConell 2007; Wadley et al. 2008). Tissue lysates were incubated on ice for 20 min, centrifuged at 16,000 *g* for 15 min and the supernatant collected for analysis. Protein concentration was determined using a bicinchoninic acid (BCA) protein assay (Pierce, Rockford, IL) with bovine serum albumin (BSA) as the standard.

For determination of mitochondrial biogenesis markers, muscle lysates were solubilized in Laemmli sample buffer and separated by SDS-PAGE. Protein was electrotransferred from the gel to PVDF membranes and blots were probed with anti-PGC-1*α* rabbit polyclonal (Calbiochem, Darmstadt, Germany), anti-mtTFA rabbit polyclonal (GenWay Biotech, San Diego, CA), anti-cytochrome c oxidase IV mouse monoclonal (Invitrogen Carlsbad, CA), and anti-cytochrome c mouse monoclonal antibodies (BD Bioscience Pharmigen, San Diego, CA) (Wadley and McConell 2007; Wadley et al. 2008; Laker et al. 2011). As a loading control, blots were reprobed with antitubulin mouse monoclonal antibody (Sigma, St. Louis, MO). The following morning, the membrane was incubated with a fluorescent secondary anti-rabbit IgG IRDye 800 nm (1:5000 dilution; Rockland, Gilbertsville, PA) or IRDye^–^ 680-conjugated anti-mouse IgG (Molecular Probes, Eugene, OR) to detect primary antibody binding. The Odyssey infrared imaging system and computer software (LI-COR Biosciences, Lincoln, NE) were used to scan the membranes for detection of the fluorescently labeled antibodies and data were expressed as integrated intensity (Wadley et al. 2008; Laker et al. 2012).

### Statistical analyses

Data obtained from male and female offspring were analyzed separately using a one-way ANOVA with Student–Newman–Kuels post hoc analysis. The IPGTT and IC data were analyzed using a two-way repeated measures ANOVA with time and group as the factors (SPSS Inc., Chicago, IL). Following observation of a significant interaction between groups, a Student–Newman–Kuel post hoc analysis was performed on each time point. All data are expressed as means ± SEM with *n* representing the number of animals per litter from each group. *P *<* *0.05 was considered statistically significant.

## Results

### Growth profile and body and organ weights

**Figure 1:**
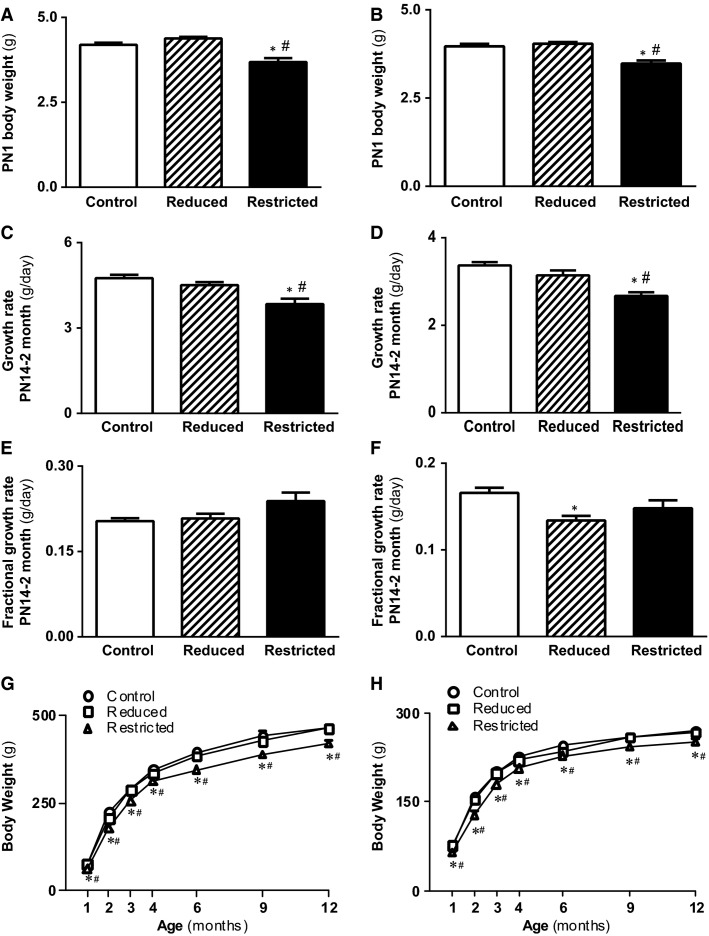
Body weights and growth trajectory in male and female offspring. Body weights were measured at PN1 (A, B), growth rates between PN14 and 2 months (absolute [C, D] and fractional [E, F]), and body weights from 1 to 12 months for determination of growth trajectory (G, H) in male and female Control, Reduced, and Restricted offspring. Values are expressed as means ± SEM; *n *=* *8–15 per group. *P <* *0.05 versus Control, ^*#*^*P *<* *0.05 versus Reduced.

Uteroplacental insufficiency reduced total (male and female) F1 litter size (5.2 ± 0.3 Restricted pups vs. 8.7 ± 0.5 Control pups, data not shown) and average litter (male and female) birth weight by 15–16% compared with sham-operated Controls at PN1 (*P *<* *0.05, Fig. 1A and B). Restricted male and female offspring had reduced peripubertal absolute growth rate (*P *<* *0.05, Fig. 1C and D) with no changes in fractional growth rate (Fig. 1E and F) from PN14 to 2 months. Subsequently, Restricted male and female offspring had reduced body weights from birth to postmortem at 12 months of age compared with Control and Reduced (*P *<* *0.05, Fig. 1G and H). Reducing litter size in mothers exposed to sham surgery at PN1 to match that of Restricted litters (five pups per litter) did not alter body weights from birth through 12 months, however, fractional growth rate in females from PN14 to 2 months was reduced compared with offspring born to sham-operated mothers with unchanged litter size (*P *<* *0.05, Fig. 1A–H). Despite the reductions in body weights of Restricted offspring, relative heart, left ventricle, kidney, pancreas, and dorsal fat weights at postmortem were not different between groups of male and female offspring (Table 1). Relative gastrocnemius weights were significantly higher in Reduced and Restricted compared with Control (*P *<* *0.05, Table 1), while in females but not males, relative liver weights were lower in Restricted compared with Control and Reduced (*P *<* *0.05, Table 1).

**Table 1:** Relative organ weights in 12-month-old male and female offspring

	Control	Reduced	Restricted
Male body weight (g)	462 ± 8	461 ± 8	481 ± 10*†
Male organ weights (% body weight)			
Heart	0.306 ± 0.003	0.306 ± 0.002	0.306 ± 0.003
Left ventricle	0.224 ± 0.002	0.223 ± 0.001	0.222 ± 0.002
Kidney	0.543 ± 0.006	0.540 ± 0.005	0.540 ± 0.006
Pancreas	0.165 ± 0.006	0.162 ± 0.009	0.152 ± 0.007
Liver	2.519 ± 0.027	2.541 ± 0.036	2.636 ± 0.032
Gastrocnemius	0.373 ± 0.003	0.394 ± 0.006*	0.397 ± 0.006*
Dorsal fat	1.755 ± 0.121	1.708 ± 0.127	1.622 ± 0.074
Female body weight (g)	269 ± 3	267 ± 5	260 ± 6*†
Female organ weights (% body weight)			
Heart	0.357 ± 0.002	0.358 ± 0.002	0.361 ± 0.003
Left ventricle	0.267 ± 0.003	0.262 ± 0.003	0.269 ± 0.003
Kidney	0.608 ± 0.008	0.625 ± 0.016	0.598 ± 0.006
Pancreas	0.249 ± 0.009	0.234 ± 0.008	0.240 ± 0.017
Liver	2.860 ± 0.025	2.874 ± 0.008	2.833 ± 0.022*†
Gastrocnemius	0.399 ± 0.008	0.429 ± 0.007*	0.425 ± 0.009*
Dorsal fat	2.059 ± 0.073	1.987 ± 0.100	1.884 ± 0.120

Weights of heart, left ventricle, kidney, pancreas, liver, and gastrocnemius relative to body weights were measured at postmortem in male and female Control, Reduced, and Restricted offspring. Values are expressed as means ± SEM; *n *=* *10–15 per group.

* *P *<* *0.05 versus Control, ^†^*P *<* *0.05 versus Reduced.

### Systolic blood pressure, food and water intake, and renal function

**Figure 2:**
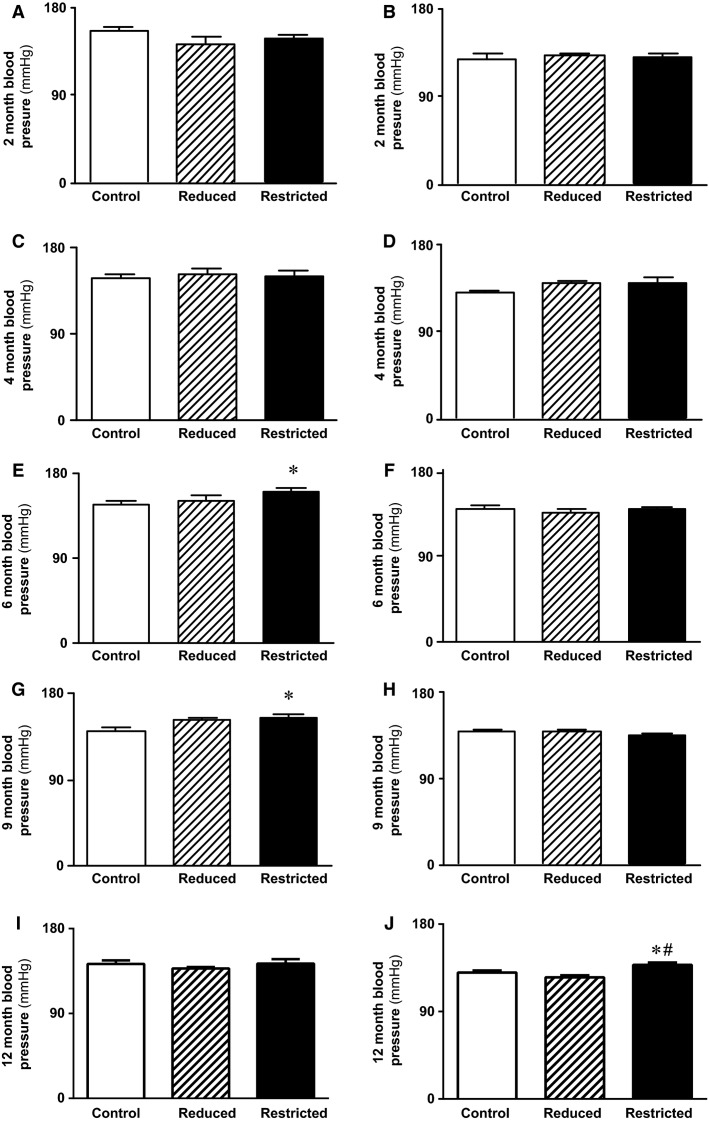
Systolic blood pressure in male and female offspring. Blood pressure was measured via a tail cuff at 6 months (A, B), 9 months (C, D), and 12 months (E, F) in male and female Control, Reduced, and Restricted offspring. Values are expressed as means ± SEM; *n *=* *8–15 per group. *P <* *0.05 versus Control; ^#^*P *<* *0.05 verses Reduced.

Systolic blood pressure was not different between Control and Reduced male and female offspring at 2, 4, 6, 9, and 12 months of age (Fig. 2A–J). In Restricted male offspring, blood pressure was comparable to Controls at 2 and 4 months (Fig. 2A and C), however, blood pressure was elevated at 6 (+13 mmHg, *P *<* *0.05) and 9 months (+14 mmHg, *P *<* *0.05, Fig. 2E and G) which was not evident at 12 months of age (Fig. 2I). In females, blood pressure in Restricted was not different to Controls at 2, 4, 6, and 9 months of age (Fig. 2B, D, F, and H), but was increased by 8 mmHg at 12 months (+6%, *P *<* *0.05, Fig. 2J) compared to Control.

In males but not females, Restricted consumed on average 15% more food compared with Reduced over 24 h (*P *<* *0.05, Table 2). Similarly, Restricted males consumed more water than Reduced males (+16%, *P *<* *0.05, Table 2). Urinary output, measured by urine production over 24 h, and excretions of albumin, creatinine, total protein, and glucose was not different between groups (Table 2). In females, urinary output was greater in Restricted (+29%) compared with Control (*P *<* *0.05, Table 2). Reduced females had greater urinary potassium (+31%) and chloride excretion (+30%) compared with Control females (*P *<* *0.05, Table 1). Urinary glucose excretions were greater in Reduced versus Restricted females (*P *<* *0.05), while urinary creatinine was greater in Reduced compared with Control and Restricted females (*P *<* *0.05, Table 2).

**Table 2:** Twenty-four-hour food and water intake and urinary excretions in 12-month-old male and female offspring

	Control	Reduced	Restricted
Males			
Food intake (g/24 h/kg)	42 ± 1	39 ± 1	46 ± 2†
Water intake (mL/24 h/kg)	72 ± 4	66 ± 4	79 ± 2†
Urine production (L/24 h/kg)	0.036 ± 0.002	0.031 ± 0.002	0.039 ± 0.004
Glucose (mmol/L/24 h/kg)	0.043 ± 0.005	0.044 ± 0.006	0.029 ± 0.008
Creatinine (mmol/L/24 h/kg)	0.090 ± 0.013	0.099 ± 0.011	0.104 ± 0.021
Total protein (mg/L/24 h/kg)	16.924 ± 3.105	20.547 ± 2.596	23.148 ± 5.152
Albumin (mg/L/24 h/kg)	0.879 ± 0.175	1.031 ± 0.177	0.831 ± 0.199
Sodium (mmol/L/(24 h)/kg)	0.648 ± 0.051	0.844 ± 0.081	0.693 ± 0.081
Potassium (mmol/L/(24 h)/kg)	1.766 ± 0.152	1.784 ± 0.140	1.568 ± 0.185
Chloride (mmol/L/(24 h)/kg)	2.118 ± 0.137	2.220 ± 0.161	2.050 ± 0.185
Females			
Food intake (g/24 h/kg)	64 ± 2	65 ± 3	65 ± 4
Water intake (mL/24 h/kg)	141 ± 7	134 ± 6	159 ± 11
Urine production (L/24 h/kg)	0.064 ± 0.005	0.075 ± 0.004	0.091 ± 0.009*
Glucose (mmol/L/24 h/kg)	0.023 ± 0.003	0.032 ± 0.006	0.012 ± 0.003†
Creatinine (mmol/L/24 h/kg)	0.081 ± 0.012	0.125 ± 0.016*	0.066 ± 0.012†
Total protein (mg/L/24 h/kg)	6.570 ± 0.857	8.906 ± 2.024	6.029 ± 1.096
Albumin (mg/L/24 h/kg)	0.443 ± 0.090	0.448 ± 0.051	0.621 ± 0.090
Sodium (mmol/L/(24 h)/kg)	1.389 ± 0.179	1.891 ± 0.119	1.508 ± 0.314
Potassium (mmol/L/(24 h)/kg)	2.975 ± 0.307	4.296 ± 0.356^*^	2.982 ± 0.345†
Chloride (mmol/L/(24 h)/kg)	3.743 ± 0.312	5.352 ± 0.381*	4.239 ± 0.500

Food and water intake and urinary glucose, creatinine, total protein, albumin, and electrolytes (sodium, potassium, and chloride) were measured over 24 h in male and female Control, Reduced, and Restricted offspring. Values are expressed as means ± SEM; *n *=* *10 per group).

* *P *<* *0.05 versus Control, ^†^*P *<* *0.05 versus Reduced.

### Glucose tolerance, insulin secretion, whole body insulin sensitivity, appetite-regulating hormone concentrations, and mitochondrial biogenesis

**Figure 3:**
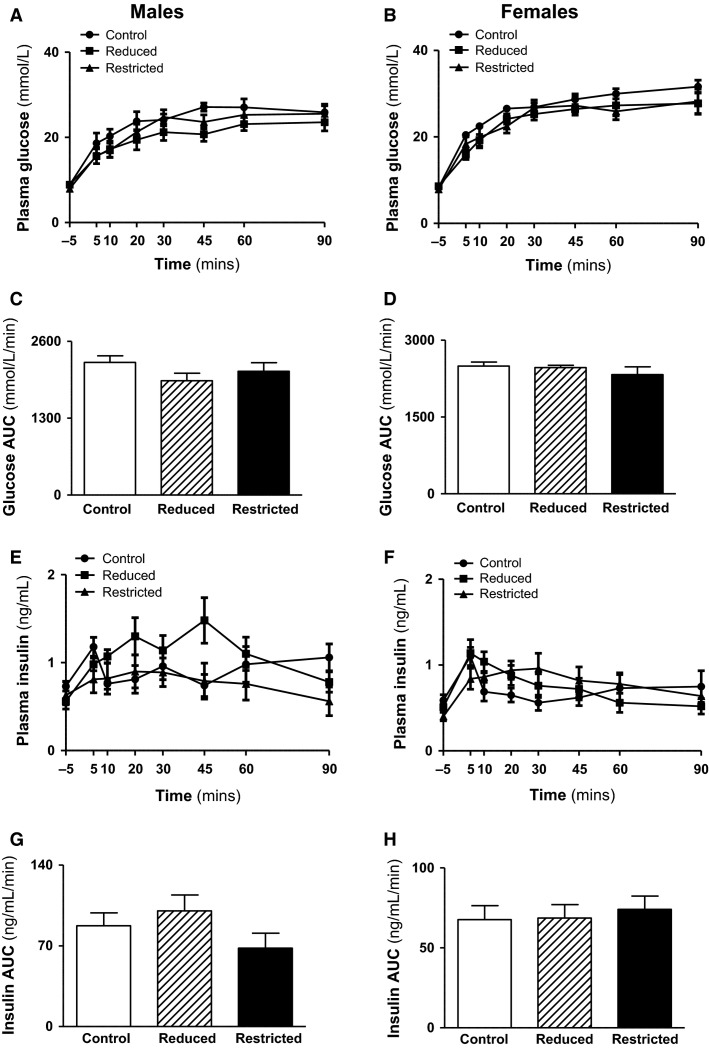
Plasma glucose and insulin prior to and in response to IPGTT. Plasma glucose and insulin were measured in samples taken at 0, 5, 10, 20, 30, 45, 60, and 90 min during the IPGTT. Plasma glucose levels in response to a glucose load (A, B), glucose AUC (C, D), plasma insulin levels in response to a glucose load (E, F), and insulin AUC (G, H) in male and female Control, Reduced, and Restricted offspring. Values are expressed as means ± SEM; *n *=* *9–10 per group. AUC, area under the curve; IPGTT, intraperitoneal glucose tolerance test.

**Figure 4:**
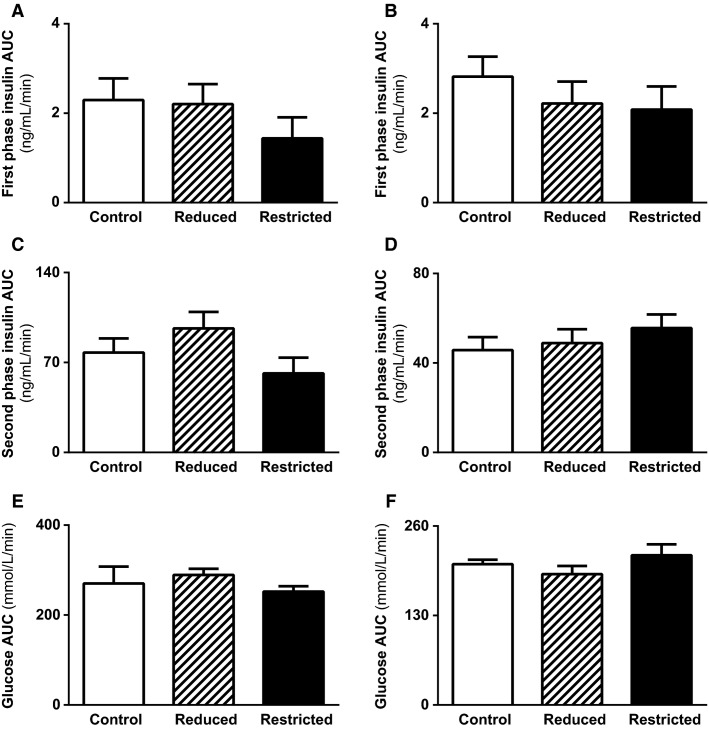
Insulin secretion and whole body insulin sensitivity in male and female offspring. Insulin secretion was measured in response to a glucose load. First-phase (A, B), second-phase (C, D) insulin AUC and whole body insulin sensitivity as measured by the glucose AUC in response to IC (E, F) in male and female Control, Reduced, and Restricted offspring. Values are expressed as means ± SEM; *n *=* *7–10 per group. AUC, area under the curve; IC, insulin challenge.

**Figure 5:**
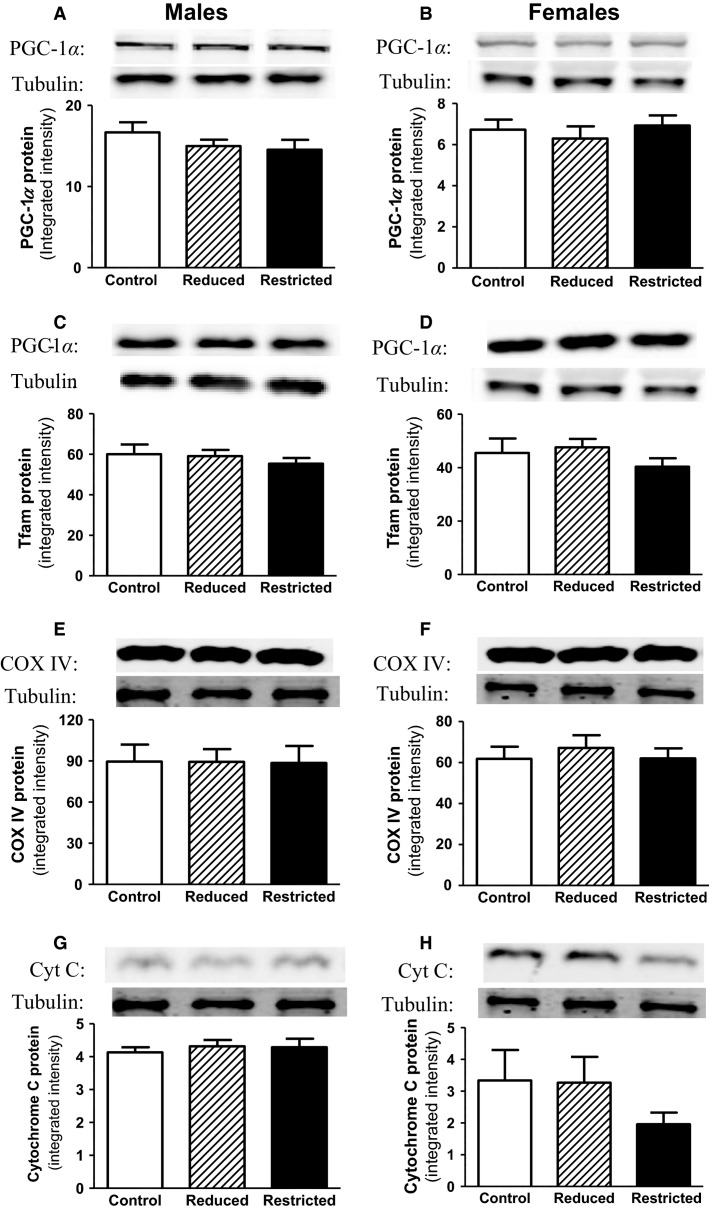
Skeletal muscle mitochondrial biogenesis markers. Mitochondrial biogenesis markers, PGC-1*α*, Tfam, COX IV, and cytochrome c protein levels were measured in gastrocnemius muscle by immunoblotting in male and female Control, Reduced, and Restricted offspring. Values are expressed as means ± SEM; *n *=* *8–10 per group. Peroxisome proliferator-activated receptor (PPAR)-*γ* coactivator-1*α* (PCG-1*α*), cytochrome c oxidase IV (COX IV).

Fasting plasma insulin and glucose concentrations and fasting insulin to glucose ratio were not different between Control, Reduced, and Restricted groups in both male and female offspring (Table 3). Similarly HOMA-IR, a measure of hepatic insulin sensitivity, was not different between groups in males (Table 3). In females, however, HOMA-IR was reduced in Restricted (-35%) compared with Control and Reduced (*P *<* *0.05, Table 3). Circulating plasma leptin concentrations were higher in Reduced males compared with Control and Restricted (*P *<* *0.05, Table 3), but no differences were observed in female offspring. Plasma ghrelin and adiponectin concentrations were not different across groups in both male and female offspring (Table 3). However, there was a trend toward a reduction in ghrelin in Reduced and Restricted females compared to Control (*P *=* *0.082 and 0.087, respectively). In response to an IPGTT, plasma glucose and insulin concentrations were not different between groups (Fig. 3A, B and E, F) as evident by the glucose and insulin AUC (Fig. 3C, D and G, H). First- and second-phase insulin secretion was not different between groups (Fig. 4A–D). Whole body insulin sensitivity, assessed by the glucose area under curve in response to an insulin challenge, was also not different between groups in both male and female offspring (Fig. 4E and F). Protein expression of mitochondrial biogenesis markers (PGC1*α*, Tfam, COX IV, and cytochrome c) was not different between groups in both male and female offspring (Fig. 5A–H).

**Table 3:** Basal metabolic parameters in 12-month-old male and female offspring

	Control	Reduced	Restricted
Males			
Fasting insulin (ng/mL)	0.73 ± 0.05	0.56 ± 0.04	0.63 ± 0.16
Fasting glucose (mmol/L)	8.85 ± 0.40	8.87 ± 0.20	7.95 ± 0.37
Fasting insulin:glucose ratio	0.084 ± 0.007	0.058 ± 0.008	0.087 ± 0.022
HOMA-IR	6.86 ± 0.59	5.24 ± 0.41	5.53 ± 1.47
Leptin (pg/mL)	7252 ± 494	14,419 ± 808*	7890 ± 862†
Adiponectin (ng/mL)	6685 ± 507	7891 ± 1439	6693 ± 512
Ghrelin (ng/mL)	9.20 ± 1.36	10.57 ± 1.20	9.89 ± 1.17
Females			
Fasting insulin (ng/mL)	0.59 ± 0.07	0.51 ± 0.06	0.40 ± 0.05
Fasting glucose (mmol/L)	8.32 ± 0.40	8.57 ± 0.33	7.87 ± 0.46
Fasting insulin:glucose ratio	0.072 ± 0.009	0.052 ± 0.008	0.053 ± 0.007
HOMA-IR	5.20 ± 0.61	5.26 ± 0.47	3.36 ± 0.41*†
Leptin (pg/mL)	4096 ± 483	4067 ± 475	3738 ± 295
Adiponectin (ng/mL)	7427 ± 690	10,164 ± 1402	7428 ± 708
Ghrelin (ng/mL)	12.41 ± 1.77	7.24 ± 1.79	8.27 ± 0.72

Fasting plasma glucose and insulin, ratio of fasting insulin to glucose, HOMA-IR, and plasma leptin, ghrelin, and adiponectin concentrations were measured in male and female Control, Reduced, and Restricted offspring. Values are expressed as means ± SEM; *n *=* *9–10 per group).

* *P *<* *0.05 versus Control, ^†^*P *<* *0.05 versus Reduced.

### Retinal function

**Figure 6:**
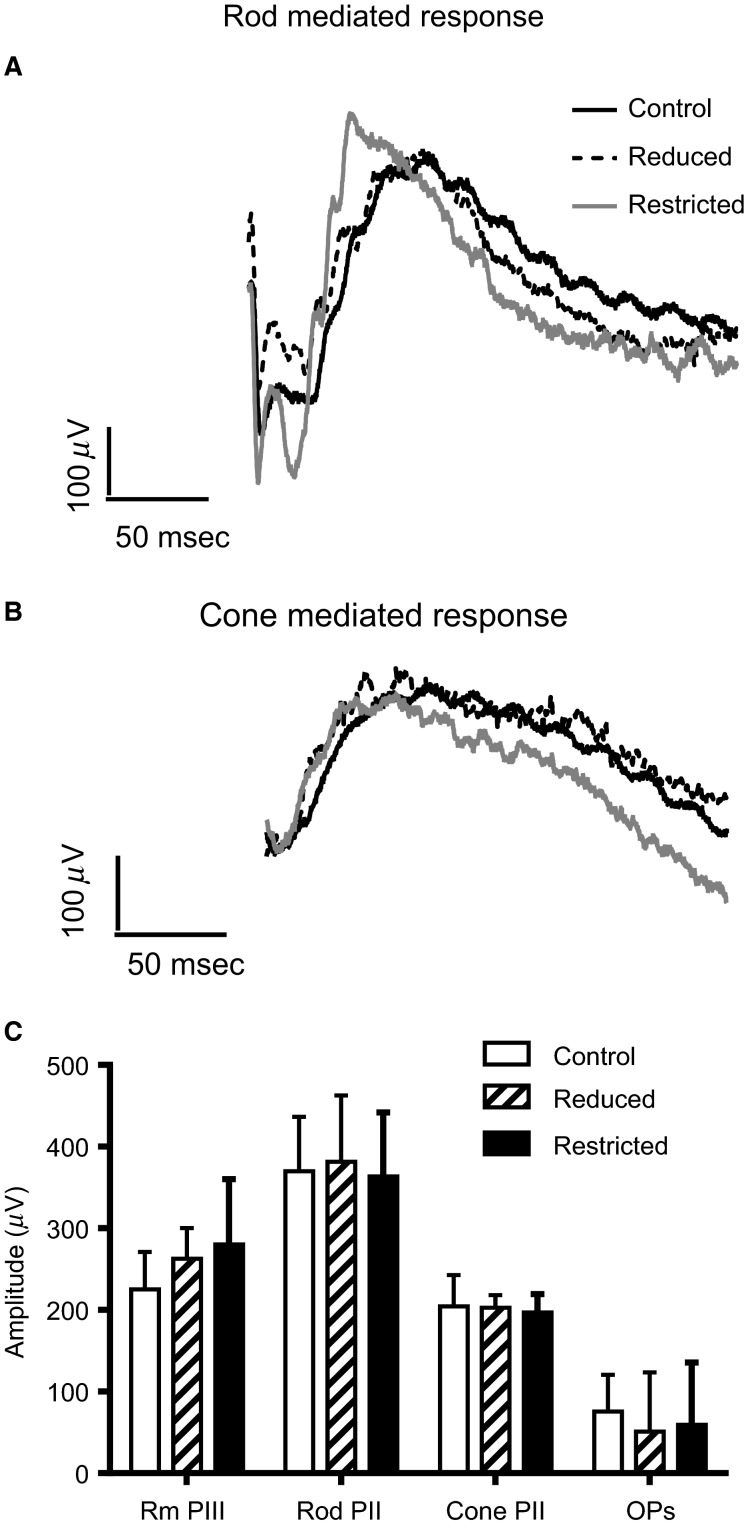
Retinal function. Representative waveforms of rod-mediated (A) and cone-mediated (B) retinal function in Control, Restricted, and Reduced male rats. Graph showing the average a-wave (rod PIII), b-wave (rod PII), summed Ops, and cone b-wave (Cone PII) in Control, Reduced, and Restricted rats (C). No difference in amplitude was detected in any of the ERG parameters measured.

Retinal function was assessed in Control, Reduced, and Restricted male offspring using the flash ERG. Figure 6A and B show representative waveforms of rod-mediated and cone-mediated retinal function. Very little difference was observed in the waveforms generated from the three groups (Fig. 6A and B). There were no differences in the amplitude of photoreceptor (a-wave) or inner retinal (b-wave and OPs) function or in cone-mediated function (cone b-wave) in Control, Reduced, and Restricted litter male offspring (Fig. 6C). Timing of each of the waveforms was also no different across the three groups (data not shown).

## Discussion

The main findings of this study was that growth-restricted females developed elevated blood pressure with advanced age, while this effect was not evident in Restricted males at 12 months, despite transient hypertension at 6 and 9 months of age. Furthermore, reduced HOMA was reported in Restricted females compared with Controls, indicating enhanced hepatic insulin sensitivity (Kahn et al. 2008; Henquin 2009). In contrast to the previous study in 6-month-old rats (Wadley et al. 2008), skeletal muscle mitochondrial biogenesis markers were not reduced in Restricted offspring compared to Controls. Furthermore, contrary to our hypothesis, there was no significant change in renal and metabolic function in male growth-restricted rats at 12 months. Thus, in our cohort, aging normalizes the impaired glucose tolerance and reduced mitochondrial biogenesis in males that was initially reported at 6 months of age (Wadley et al. 2008). However, in 12-month-old females, uteroplacental insufficiency resulted in elevated blood pressure and reduced urinary output but demonstrate enhanced hepatic insulin sensitivity.

In the current study, Restricted males and females were significantly smaller at birth and this reduction was comparable to studies in rodent (O’Dowd et al. 2008; Wadley et al. 2008; Wlodek et al. 2008) and humans (Barker et al. 1989; Wollmann 1998). Consistent with some (Laker et al. 2011; Moritz et al. 2009; Gallo et al. 2012a, 2013; Tran et al. 2012, 2013) but not all studies (Mazzuca et al. 2010; Gallo et al. 2012b), Restricted offspring remained smaller, with no evidence of catch-up growth to Control levels. The inconsistencies between studies may be due to the degree of growth restriction at birth that may impact on metabolic and renal outcomes differently across studies. Indeed, the absence of accelerated catch-up growth may protect these growth-restricted offspring from independently associated disease outcomes (Eriksson et al. 1999). Certainly, in the Restricted male and female offspring, there was no significant change in the plasma concentrations of leptin or adiponectin, suggesting that these hormones are not modulating the growth of the offspring. However, it is well established that leptin transport across the blood–brain barrier is essential for the maintenance of normal body weight (Burguera et al. 2000). Thus, altered transport of appetite-regulating hormones would reduce their efficiency, which may lead to the reduced body weight in the Restricted offspring. The expression of key transporters in the brain therefore requires further investigation.

While many studies normalize their control group to the litter size of the Restricted, the current study controlled for the reduction in litter size by including a reduced litter group and a nonreduced litter (Control) group. Reducing litter size after birth on PN1 did result in alterations in body weight compared to Restricted offspring, with growth trajectory in the Reduced cohort similar to Controls. This was in contrast to other studies (O’Dowd et al. 2008; Wadley et al. 2008; Wlodek et al. 2008), which reported catch-up growth, thus it is difficult to draw comparisons to the previous 6-month cohort (O’Dowd et al. 2008; Wadley et al. 2008; Wlodek et al. 2008).

Some human studies have reported that reduced mitochondrial biogenesis markers are associated with development of type 2 diabetes (Kelley et al. 2002; Petersen and Shulman 2002). However, there is little evidence in low birth weight human studies to support skeletal muscle mitochondrial dysfunction as being a primary defect in the development of insulin resistance (Brons et al. 2008, 2010, 2012). At 6 months of age, previous studies have shown growth-restricted male rats had reduced expression of several skeletal muscle mitochondrial biogenesis markers, including PGC-1*α*, compared with Controls, despite relative (to body weight) muscle mass being unaltered (Wadley et al. 2008; Laker et al. 2012). The present study, however, reported no differences in protein expression of PGC-1*α* or other mitochondrial biogenesis markers across the experimental groups at 12 months of age. The present study also found relative gastrocnemius weight was significantly higher in the Restricted and Reduced 12 months old rats compared to Controls. Collectively, the findings from the present study and our previous studies (Wadley et al. 2008; Laker et al. 2012) suggest an association between lower skeletal muscle PGC-1*α* levels and a reduction in relative muscle mass as a consequence of catch-up growth. Furthermore, in addition to its role in mitochondrial biogenesis, increased PGC-1*α* has recently been linked to the maintenance of skeletal muscle mass through regulation of autophagy and proteasome degradation (Cannavino et al. 2014). Further studies are now required to establish if this observed association between skeletal muscle PGC-1*α* levels and lean muscle mass is causal in our model.

Aging is a secondary factor that could contribute to the development of insulin resistance and impaired glucose tolerance in adulthood. Indeed others have reported that insulin sensitivity and glucose tolerance declines with age (DeFronzo 1981; Reaven et al. 1983; Chen et al. 1988). A study has reported that insulin resistance develops from 9 months of age in rodents. It is closely linked with the decline in mitochondrial function (Karakelides et al. 2010) due to an accumulation of mtDNA faults caused by increased reactive oxygen species (Huang and Hood 2009). However, age-related increases in adiposity were a more significant factor in development of insulin resistance (Karakelides et al. 2010). Importantly, the Restricted offspring in this study were smaller than the Controls at 12 months, with no differences in adiposity or lean mass. In Restricted females, hepatic insulin sensitivity was significantly higher at 12 months of age compared to Controls, however this could potentially be due to the absence of catch-up growth. A similar association has been reported in a previous study (Tran et al. 2012, 2013) and in 32-week-old rats malnourished in utero and during 2 weeks of lactation (Lim et al. 2011). The authors postulated that if growth-restricted offspring were maintaining their growth trajectory similar to that of in utero growth, in the absence of catch-up growth, they are programmed for improved postnatal glucose metabolism. These findings, along with ours, support previous studies that growth-restricted rats had significantly better glucose tolerance and insulin sensitivity than controls in late adulthood provided there is no catch-up growth (Ozanne et al. 1996; Shepherd et al. 1997). Furthermore, the glucose tolerance of 6-month-old growth-restricted rats did not differ between experimental groups (Wadley et al. 2008), however this study also reported increased HOMA in Restricted female, but not male offspring (Wadley et al. 2008). Further to this, our study was the first to examine retinal function in a model of growth restriction. Contrary to our hypothesis, retinal function was not impaired, and this is likely to be due to a lack of hyperglycemia in our model (Phipps et al. 2007).

These findings suggest that additional factors, such as obesity, in combination with aging may be necessary to unmask insulin resistance and changes in glucose tolerance in rats exposed to both pre- and postnatal growth restriction. Of interest, in our cohort, there was a trend toward a decrease of ghrelin in the Restricted and Reduced females. Ghrelin, an orexigenic factor that controls energy balance and food intake is associated with glucose metabolism (Darendeliler et al. 2008). This suggests that in our Restricted and Reduced females there may be a trend toward an increased risk of developing obesity and glucose intolerance, however, a more detailed characterization of the pathways responsible for energy and glucose maintenance are required.

Consistent with our previous study (Gallo et al. 2012a,b), Restricted females had no changes in systolic blood pressure at 6 month, however unlike our previous study elevations in systolic blood pressure was reported at 12 months. This was associated with increased urinary flow rate but urinary metabolite excretions were comparable across groups. Indeed, the hypertensive phenotype and increased urinary excretion may be associated with alterations in kidney nephron number or expression of various genes involved in the renin–angiotension system. Furthermore, there may be programmed age-dependent rise in glomerular damage and apoptosis in the current study and further investigation is warranted. Similarly to our previous study (Wlodek et al. 2008), male Restricted offspring had elevations in systolic blood pressure at 6 months which persisted to 9 months (this study) compared to Controls, but no changes were observed at 12 months. While elevations in systolic blood pressure at 6 and 9 months of age in growth-restricted male rats may contribute to end-organ damage, there appears to be no changes in renal function at 12 months of age, suggesting maintenance of glomerular integrity. It is possible, however, that growth-restricted male offspring at 12 months of age, exposed to a high-salt/fat diet or other lifestyle challenges may increase the risk of developing renal dysfunction through alterations in pathways essential for normal homeostatic regulation of kidney function.

Contrary to the hypothesis, females had improved insulin sensitivity but elevated blood pressure at 12 months of age, while male offspring demonstrate comparable glucose tolerance to Controls but developed transient hypertension at 6 and 9 months of age. Furthermore, the expression of the mitochondrial biogenesis markers were not reduced in male and female Restricted offspring, as was reported in the 6-month study (Wadley et al. 2008; Laker et al. 2012) and this is possibly due to the higher lean muscle mass in the Restricted and Reduced rats by 12 months of age. Certainly in this cohort, appetite-regulating hormones in the Restricted offspring were not significantly different and the growth trajectory was unaltered. “Catch-up growth” in growth-restricted offspring programs alterations in metabolic function (Berends et al. 2013), and in our cohort, a lack of “catch-up growth” may have played an important role in determining later disease outcomes. However, as described previously, additional secondary factors such as exposure to high-salt or high-fat diet, or further advanced age may reveal a clinically relevant phenotype that was not initially reported at 12 months of age in rats exposed to uteroplacental insufficiency and born small.
